# Role of Antioxidants in the Protection from Aging-Related Diseases

**DOI:** 10.1155/2019/7450693

**Published:** 2019-01-08

**Authors:** Daria Maria Monti, Maria Manuela Rigano, Simona Maria Monti, Herbenya Silva Peixoto

**Affiliations:** ^1^Department of Chemical Sciences, University of Naples Federico II, Complesso Universitario Monte Sant'Angelo, via Cinthia 4, 80126 Naples, Italy; ^2^Department of Agricultural Sciences, University of Naples Federico II, Via Università 100, 80055 Portici (Naples), Italy; ^3^Institute of Biostructures and Bioimaging, National Research Council, Naples 80134, Italy; ^4^Faculty of Pharmacy, Universidade Paulista UNIP, Campus Manaus BR, Av. Mário Ypiranga, 4390-Parque 10 de Novembro, 69050-030 Manaus, AM, Brazil

In recent years, oxidative stress has been considered an important factor in the pathogenesis and development of lifestyle-related diseases. In particular, the increase in reactive oxygen species (ROS) has been related to the onset and progression of human aging. ROS can be generated by normal metabolic activity as well as by lifestyle factors. Although many epidemiological studies have shown that the adoption of a healthy diet is mandatory, the molecular mechanisms by which nutrients exert antioxidant effects remain poorly understood. Concurrently, oxidation damage can also depend on inherited or acquired defects in enzymes involved in the redox-mediated signaling pathways. The resulting redox-unbalance leads to increased susceptibility towards oxidative aging-related pathologies. For these reasons, the investigation of the role of cellular antioxidant scavenger enzymes, active towards oxidative stress, is gaining increased attention.

In this special issue, we invited researchers to shed light on the molecular mechanisms involved in fighting aging-related diseases by antioxidant molecules which are able to promote healthy aging and counteract oxidative stress.

In the review from B.A.Q. Gomes et al., they analyzed the possibility to counteract Alzheimer's disease, a severe neurodegenerative disorder, whose insurgence is related to age and presents high levels of oxidative stress. The authors reported that resveratrol is able to inhibit A*β* peptide aggregation. Resveratrol is a well-known phenol endowed with antioxidant and anti-inflammatory activity, and it plays also an important role in neuronal differentiation through the activation of silent information regulator-1; thus, it can be considered as a promising tool in disease prevention.

Moreover, in the context of neurodegeneration, the anti-inflammatory mechanisms of several phytochemicals, such as curcumin, resveratrol, propolis, polyunsaturated fatty acids (PUFAs), and ginsenosides, have been extensively studied in the review from J. Wang et al. In particular, the authors reported that these phytochemicals are able to modulate and suppress neuroinflammation of the brain by different mechanisms of action. Therefore, some phytochemicals can represent a useful tool to counteract systemic inflammation and oxidative stress related to neurodegenerative diseases.

Still in the context of neurodegeneration, L.A. Ramos-Chávez et al. reported a study, on 77 women over the age of 50, on the relation between the changes in Trp catabolism and cognitive impairment associated with age. The authors hypothesize using circulating Trp levels as a new biomarker for cognitive impairment.

In the review from A. Perrelli et al., the role of avenanthramides (from *Avena sativa*) in fighting age-related diseases was deeply analyzed. Interestingly, avenanthramides have been identified as therapeutic candidates for the treatment of a cerebrovascular disorder. Finally, by using proteomic approaches, the authors found distinctive molecular pathways and redox protein modifications associated with avenanthramide activity.

The paper from Y. Ma et al. was on the protective effect and mechanism of action of an ethanol extract from the leaves of *Diospyros kaki*. The extract was used on mice treated with D-galactose, known to mimic aging in animal models. The authors found that the extract, rich in flavonoids, decreased oxidative stress levels and inflammatory mediators, restored memory impairment, and ameliorated the impairment of synaptic-related proteins.

The role and molecular mechanism of action of proteins containing reactive sulfhydryl groups involved in the response to oxidative stress has been clearly described by A. Di Fiore et al., with a special focus on carbonic anhydrases III and VII. These enzymes have been found to play an important role in cells in which oxidative stress is activated. Interestingly, both proteins are mainly localized in tissues characterized by a high rate of oxygen consumption and contain, on their molecular surface, two reactive cysteine residues eventually undergoing S-glutathionylation.

As for plant secondary metabolites, their role has been deeply analyzed in the review by G. Petruk et al., with a special focus on skin photoaging. Phenolic compounds, polyphenols, and carotenoids have been analyzed for their antiaging properties related to the reduction of oxidative stress pathways.

Finally, L. Di Renzo et al. analyzed the role of the Mediterranean diet in preventing noncommunicable diseases and, in particular, the role of wine. They found that the association of low/moderate intake of alcohol beverages, with nutraceutical-proven effectiveness and ethanol, in association with a Mediterranean diet, could determine a reduction of atherosclerosis risk onset through a positive modulation of antioxidant gene expression helping in the prevention of inflammatory and oxidative damages.

Thus, we hope that readers will find these contributions interesting and stimulating for the next coming research. We believe that these reviews will add knowledge in the correlation between oxidative stress and antioxidants in age-related pathologies.

